# Inhibition of UBE2D3 Expression Attenuates Radiosensitivity of MCF-7 Human Breast Cancer Cells by Increasing hTERT Expression and Activity

**DOI:** 10.1371/journal.pone.0064660

**Published:** 2013-05-31

**Authors:** Wenbo Wang, Lei Yang, Liu Hu, Fen Li, Li Ren, Haijun Yu, Yu Liu, Ling Xia, Han Lei, Zhengkai Liao, Fuxiang Zhou, Conghua Xie, Yunfeng Zhou

**Affiliations:** 1 Hubei Cancer Clinical Study Center, Hubei Key Laboratory of Tumor Biological Behaviors, Zhongnan Hospital, Wuhan University, Wuhan, China; 2 Department of Radiation Oncology & Medical Oncology, Zhongnan Hospital, Wuhan University, Wuhan, China; University of Navarra School of Medicine and Center for Applied Medical Research (CIMA), Spain

## Abstract

The known functions of telomerase in tumor cells include replenishing telomeric DNA and maintaining cell immortality. We have previously shown the existence of a negative correlation between human telomerase reverse transcriptase (hTERT) and radiosensitivity in tumor cells. Here we set out to elucidate the molecular mechanisms underlying regulation by telomerase of radiosensitivity in MCF-7 cells. Toward this aim, yeast two-hybrid (Y2H) screening of a human laryngeal squamous cell carcinoma radioresistant (Hep2R) cDNA library was first performed to search for potential hTERT interacting proteins. We identified ubiquitin-conjugating enzyme E2D3 (UBE2D3) as a principle hTERT-interacting protein and validated this association biochemically. ShRNA-mediated inhibition of UBE2D3 expression attenuated MCF-7 radiosensitivity, and induced the accumulation of hTERT and cyclin D1 in these cells. Moreover, down-regulation of UBE2D3 increased hTERT activity and cell proliferation, accelerating G1 to S phase transition in MCF-7 cells. Collectively these findings suggest that UBE2D3 participates in the process of hTERT-mediated radiosensitivity in human breast cancer MCF-7 cells by regulating hTERT and cyclin D1.

## Introduction

Radiotherapy is an important function in the treatment of breast cancer and its role has been extensively studied during the last several decades [1], [2]. Clinical studies have demonstrated a major benefit of adjuvant radiotherapy in increasing disease-free survival (DFS) and overall survival (OS) in breast cancer [2], and cellular radiosensitivity is an area of intense research in radiation oncology. In particular, chromosomes, which comprise the cellular cytogenetic information center, are one of the primary targets of radiation injury [3].

Telomeres, which are regions at the termini of chromosomes, are composed of TTAGGG repetitive DNA sequences and a variety of binding proteins [4]. Telomerase, is a ribonucleoprotein enzyme that synthesizes telomeric DNA and contributes to the maintenance of functional telomeres [5], [6]. Telomerase, which are activated in 90% of human tumor cells but are seldom activated in normal somatic cells, is composed of two components, human telomerase RNA(hTR) and hTERT. The expression of hTERT, closely correlates with telomerase activity and serves as an indicator of telomerase activation [7], [8]. Given the importance of telomerase in cellular synthesis of telomeres, their investigation in the context of cellular radiosensitivity is particularly important. Reduction of telomerase activity through inhibition of the expression of telomerase subunits has been shown to result in a decline in the ability of cells to repair DNA damage after irradiation, with a consequent increase in radiosensitivity [9], [10]. Preliminary studies in our group showed that suppression of hTERT or hTR expression increases the radiosensitivity of tumor cells by inhibiting telomerase activity [11], [12]. Although hTERT presents an attractive target for cancer therapy [13], [14], its potential radiosensitizing effects have not been previously studied.

The lysosome and ubiquitin-proteasome pathway (UPP) systems are the two primary pathways in intracellular protein degradation. The UPP functions in all tissues to maintain the quality control of cellular protein production through the degradation of misfolded, mutated or otherwise damaged proteins, or to degrade regulatory proteins to modulate basic cellular activities such as growth, metabolism, apoptosis, cell cycle and transcriptional regulation. Ubiquitination is one of the most important post-translational modifications in regulating protein degradation. The process of ubiquitination involves three classes of enzymes, E1, E2 and E3 [15]. To date, two E1 enzymes, around forty E2 enzymes and hundreds of E3 ligase have been found in humans [16], [17]. A E2 enzyme can interact with several E3 ligase and thereby affect multiple targets [18]. E3 ligase has attracted wide concentrations for its substrate selection specificity. Recent research has identified a large number of proteins involved in DNA damage repair, including ATM, H2Ax, BRCA1 and RAD51. Many of these are ubiquitin-like proteins, and it has been reported that the ubiquitin-proteasome plays an important role in the repair of DNA damage [19], [20]. Moreover, Mdm2, an E3 ligase, promotes the ubiquitination and degradation of p53 [21], suggesting that ubiquitination is associated with radiation-induced DNA damage repair. In contrast to the volume of data on E3 ligase, much less is known about the regulatory mechanisms of E2 enzymes. We previously showed that the E2 ubiquitin-conjugating enzyme E2N (UBE2N) was differentially expressed between radiosensitive human laryngeal squamous cell carcinoma (Hep2) and its radioresistant counterpart Hep2R.

To gain additional insight into the role of hTERT in radiosensitivity, we used the Y2H system to find novel hTERT-binding proteins. We identified UBE2D3, a member of the E2 family, as a hTERT-interacting protein and showed that UBE2D3 is required for hTERT activation of radiosensitivity. Our results demonstrate that E2 regulation potentially plays a part in signaling in the hTERT pathway.

## Materials and Methods

### Cell Lines, Transfections, Plasmids and Reagents

Hep2 and Hep2R were maintained by the Key Laboratory of Tumor Biological Behavior of Hubei Province. MCF-7 cells and HEK293T cells were obtained from the Cell Bank of the Chinese Academy of Science (Shanghai, China), maintained in 5% CO_2_ at 37°C in Dulbecco’s minimum essential medium (DMEM) containing 10% fetal bovine serum (FBS), 100 U/ml penicillin and 100 mg/ml streptomycin. All culture reagents were purchased from Hyclone, USA. Transfections were carried out using Lipofectamine 2000 (Invitrogen, USA) for plasmids and shRNA. SMART™ cDNA Library Construction Kit, Matchmaker™ Gold Yeast-two Hybrid System, Matchmaker Insert Check PCR Mix 2 and all yeast media were purchased from Clontech, USA. Telomerase PCR-Elisa kits were obtained from Roche, USA. The CCK-8 kit was purchased from Dongji, Japan. Antibodies (UBE2D3, hTERT, cyclin D1, β-actin) were purchased from Santa Cruz, USA. Vectors (pEGFP-C1, pdsRed-monomer-C1, pCMV-C-HA and pCMV-Tag2C) were obtained from Clontech, USA.

### cDNA Library Construction

Total Hep2R RNA was used to synthesize the first-strand cDNA and double-strand cDNA by SMART method (Clontech). The cDNA fragments were inserted into the pGADT7 vector, and recombinant phages were packaged in vitro. A small aliquot of packaged phage was used to infect DH10B Competent Cells. Titration and the positive clones were assayed by PCR.

### Vector Construction

Total RNA was extracted from MCF-7 cells and reverse-transcribed using a commercial kit (Takara). The ORF of UBE2D3 was PCR-amplified from the reverse transcription product with the following three primer pairs: pEGFP-C1-Forward(EcoRI): 5′-GGAATTCGATGGCGCTGAAACGGATTAA-3′ and pEGFP-C1-Reverse(BamHI): 5′-CACCACGGATCCTCACATGGCATACT TCTGAGTC-3′. PdsRed-monomer-C1-Forward(EcoRI): 5′-GGAATTCGATGGC GCTGAAACGGATTAA-3′ and pdsRed-monomer-C1-Reverse(BamHI): 5′-C ACCACGGATCCTCACATGGCATACTTCTGAGTC-3′. PCMV-Tag2C-Forward (BamHI): 5′-CGCGGATCCTTATGGCGCTGAAACGGATTAA-3′and pCMV- Tag2C-Reverse(EcoRI):5′-CCGGAATTCCTCACATGGCATACTTCTGAGTC-3′). The PCR product was digested with EcoRI and BamHI and cloned into pEGFP-C1, pdsRed-monomer-C1 and pCMV-Tag2C using the same two restriction enzyme sites. Insertion of the UBE2D3 ORF was confirmed by sequencing. hTERT was excised from the pBabe-hygro-hTERT vector (kindly provided by Dr. Jianmin Li of Nanjing Medical University) with SalI and EcoRI and cloned into pGBKT-T7 vector (Clontech). The same procedure was used to generate pCMV-C-HA-hTERT and pEGFP-hTERT. All restriction and modifying enzymes were supplied by Fermentas (USA) and were used according to the manufacturer’s instructions. All constructs were verified by DNA sequencing.

### RNA Interference

ShRNA duplexes designed against UBE2D3 (GenBank accession no. NM_181889.1) with the following sequences: GGCGCTGAAACGGATTAAT synthesized by Shanghai GenePharma (Shanghai, China), were incorporated into the pU6/GFP/Neo-shRNA vector (GenePharma) to make pU6/GFP/Neo-shRNA-UBE2D3. The sequence GTTCTCCGAACGTGTCACGT was used as negative control in all experiments.

### Y2H Assay

Competent yeast Gold and Y187 cells were prepared by TE/LiAC assay according to the Clontech protocol. After construction of pGBKT-hTERT, western blotting was used to detect hTERT protein expression. pGBKT-hTERT was transformed into Gold bacteria to test autoactivation and toxicity. The recombinant expression plasmid pGBKT-hTERT was transformed into competent Gold cells using a yeast transformation protocol. A single fresh, large (2–3 mm) colony of pGBKT-hTERT was inoculated into 50 ml of SD/−Trp liquid medium which was incubated with shaking (250–270 rpm) at 30°C until the OD_600_ reached 0.8 (16–20 hr). Cells were centrifuged (1,000 g for 5 min), and the supernatant discarded. The pellet was resuspended to a cell density of >1×10^8^ cells per ml in SD/−Trp (4–5 ml). A 1-ml aliquot of Hep2R cDNA library strain was thawed to room temperature in a water bath and 10 µl removed for titering on 100 mm SD/−Leu agar plates. 1 ml of Hep2R cDNA Library was combined with 4–5 ml pGBKT-hTERT in a sterile 2 L flask and 45 ml of 2xYPDA liquid medium (with 50 µg/ml kanamycin) was added. Cells from the library vial were rinsed twice with 1 ml 2xYPDA, added to the 2 L flask and incubated at 30°C for 20–24 hr with slow shaking (30–50 rpm). After 20 hr, a drop of the culture was checked under a phase contrast microscope (40X). Cells were centrifuged at 1,000 g for 10 min. Meanwhile, the 2 L flask was rinsed twice with 50 ml 0.5xYPDA (with 50 µg/ml kanamycin), rinses were combined, and this was used to resuspend the pelleted cells. Cells were centrifuged at 1,000 g for 10 min and the supernatant discarded. All pelleteted cells were resuspended in 10 ml of 0.5xYPDA/Kan liquid medium. The total volume of cells+medium was measured. From the mated culture, 100 µl of 1/10, 1/100, 1/1,000, and 1/10,000 dilutions were spread on 100 mm agar plates and incubated at 30°C for 3–5 days. The remainder of the culture was plated, 200 µl per 150 mm on DDO/X/A (50–55 plates) and incubated at 30°C for 3–5 days. The number of screened clones (diploids) was calculated by counting the colonies from the DDO plates after 3–5 days. All blue colonies that grew on DDO/X/A were patched out onto higher stringency QDO/X/A agar plates using a flat sterile toothpick or yellow pipette tip. All QDO/X/A positive interactions were further analyzed to identify duplicates and to verify that the interactions are genuine.

### Confocal Imaging Analysis of hTERT and UBE2D3 Co-localization

MCF-7 cells were grown on glass coverslips and co-transfected with pEGFP-hTERT and pdsRed-UBE2D3. After 24 hr, cells were fixed in 4% paraformaldehyde at room temperature for 10 min, mounted with Vectashield (Vector Laboratories) and visualized using a OLYMPUS 510 confocal microscope. Localization of hTERT and UBE2D3 was examined using confocal microscopy.

### Co-immunoprecipitation

Briefly, HEK293T cells were grown to 80% confluence in 10 cm dishes and then co-transfected with plasmid HA-hTERT and Flag-UBE2D3 using the transfection method described above. At 24 hr post-transfection, cells were lysed in 800 µl immunoprecipitation buffer. After a 12,000 rpm centrifugation for 15 min at 4°C, the supernatant was collected and incubated with anti-hTERT, anti-HA (Sigma) or anti-UBE2D3 anti-FLAG (Sigma), then precipitated by protein-A agarose (Merck) over night at 4°C. After washing three times with washing buffer (immunoprecipitation buffer and 500 mM NaCl), bound protein was eluted by boiling in SDS-PAGE gel loading buffer, and detected as described for western blotting. All experiments were repeated 3 times with similar results.

### Western Blotting Analysis

PshRNA-UBE2D3 and negative control were transfected into MCF-7 cells. The expression of hTERT, UBE2D3, cyclin D1 and β-actin as a loading control were determined by western blotting after 48 hr. Total protein from those cells were extracted after transfection with plasmids as indicated for 48 hr. Proteins were loaded and separated by 10% SDS gel electrophoresis and transferred to PVDF membrane. Membranes were blocked with 5% non-fat milk and 0.1% Tween for 1 hr. Blots were then probed overnight at 4°C with primary antibodies at dilutions of 1∶400 (anti-hTERT and anti-UBE2D3) and 1∶500 (anti-cyclin D1 and β-actin). After 1–2 hr incubation with horseradish peroxide-conjugated secondary antibody, immunoreactive proteins were detected by enhanced chemiluminescence using the ECL detection system per the manufacturer’s instructions (Beyotime, Shanghai, China). All experiments were repeated 3 times with similar results. The results were analyzed by Image J software.

### ELISA Assay

After 48 hr transfection with pEGFP-UBE2D3, pshRNA-UBE2D3 and pshRNA-NC, proteins were extracted by cell lysis, and the BSA method was used to assay the protein concentration. The telomerase activity of each sample was determined using the Telo-TAGGG Telomerase PCR-Elisa Kit (Roche, Switzerland) per the manufacturer’s instructions. A microplate reader (Bio-Rad, USA) was used to measure the absorbance of samples at 450 nm (with a reference wavelength of approx 690 nm) 30 min after addition of the stop reagent. Data were normalized by *Renilla* luciferase assay. Each experiment was done at least three times in triplicate wells and the significance of the differences between the means was assessed using Student's t-test.

### Cell Cycle and Cell Proliferation Assay

MCF-7 cells were transfected with pshRNA-UBE2D3 and negative control. Samples were collected at the indicated time points and fixed in 70% ethanol overnight. For cell cycle analysis, fixed cells were treated with RNase for 20 min before addition of 5 µg/mL PI and analyzed by FACS. Meanwhile, cells diluted serum-free DMEM medium, were seeded at 2×10^3^ cells/well in 96-well plates and cultured in 100 µl of culture medium. After 12 hr, 10 µl CCK-8 was added to each well and samples were then incubated at 37°C for 4 hr. The absorbance was then read at 450 nm using a 96-well plate reader. Each experiment was done at least three times in triplicate wells. Statistical analyses of data were performed using Student's t-test.

### Colony Formation Assay

An appropriate number of cells transfected with pshRNA-UBE2D3 or negative control were plated into 6-well plates. Each group of cells was irradiated with 0, 1, 2, 4, 6, 8 or 10 GY of ionizing radiation and incubated at 37°C in 5% CO_2_ for 14 days. Colonies were then fixed and stained with crystal violet (1% in absolute alcohol). Cell survival was measured by standard colony formation after radiation treatment. Colonies containing>50 cells were rated as deriving from viable, clonogenically capable cells. The data were fit into the linear-quadratic model, and the survival curve of each group was demonstrated by Graphpad prism5.0 software. Radiobiological parameters were calculated according to the survival curves. Each experiment was done at least three times in triplicate wells.

### Statistical Analysis

All of the experiments were replicated three times. Data are expressed as Mean±SD. Quantification of band densities was performed using Image J software. Statistical analysis was performed using software SPSS 19.0 and Graphpad prism5.0 software. The significance of differences between the means was assessed using Student's t-test. *P*<0.05 was considered to be statistically significant.

## Results

### Construction of Hep2R cDNA Library and Yeast Two-Hybrid Assay

**Figure 1:**
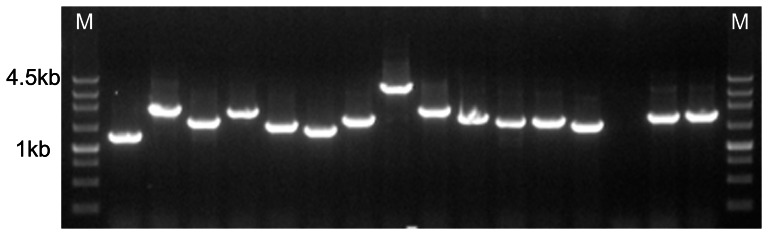
The total RNA, isolated from Human laryngeal squamous cell carcinoma radioresistant cell Hep2R, was used to synthesized the first-strand cDNA and double-strand cDNA by SMART method (Clontech). The cDNA fragments were inserted into the pGADT7 vector, and the recombinant phage were packaged in vitro. A small portion packaged phage was used to infected DH10B Competent Cells. Titration and the positive clones were assayed by PCR. Fig. 1 shows the inserted fragment of Hep2R cell full length cDNA library detected through construction electrophoresis. Table 1 shows the proteins found through Y2H from Hep2R cell cDNA library.

The total RNA we extracted from Hep2R cells had less degradation and molecules were complete. Then, double-stranded cDNA was successfully synthesized. The titer of the constructed cDNA phage expression library for Hep2R was 2.1×10^6^ pfu/mL with a recombination rate of 98.16%. Figure 1 shows the range of the fragment length of inserted cDNA was between 1.0 and 2.5 kb, with an average of 1.5 kb. On the basis of the construction of the Hep2R cell full-length cDNA library, approximately 20 proteins expressed by the library were found to interact with hTERT through the Y2H assay. (Table 1).

**Table 1:** hTERT interactors identified in Y2H library screen.

GenBank	Description
NM_014331.3	Homo sapiens solute carrier family 7(cationic amino acid transporter, y+ system) member 11 (SLC7A11), mRNA
NM_181889.1	Homo sapiens ubiquitin-conjugating enzyme E2D3 (UBC4/5 homolog, yeast) (UBE2D3/UbcH5c), transcript variant 5, mRNA
NM_001080415.1	Homo sapiens U2-associated SR140 protein (SR140), mRNA
NM_004136.2	Homo sapiens iron-responsive element binding protein 2 (IREB2), mRNA
NM_003242.5	Homo sapiens transforming growth factor, beta receptor II (70/80 kDa) (TGFBR2), transcript variant 2, mRNA
NM_000169.2	Homo sapiens galactosidase, alpha (GLA), mRNA
NM_015640.3	Homo sapiens SERPINE1 mRNA binding protein 1 (SERBP1), transcript variant 4, mRNA
NM_003746.2	Homo sapiens dynein, light chain, LC8-type 1 (DYNLL1), transcript variant 3, mRNA
NM_016018.4	Homo sapiens PHD finger protein 20-like 1 (PHF20L1), transcript variant 1, mRNA
NM_001686.3	Homo sapiens ATP synthase, H+ transporting, mitochondrial F1 complex, beta polypeptide (ATP5B), nuclear gene encoding mitochondrial protein, mRNA
NM_001743.3	Homo sapiens calmodulin 2 (phosphorylase kinase, delta) (CALM2), mRNA
NM_152266.3	Homo sapiens chromosome 19 open reading frame 40 (C19orf40), mRNA
NM_002622.4	Homo sapiens prefoldin subunit 1 (PFDN1), mRNA
NM_012073.3	Homo sapiens chaperonin containing TCP1, subunit 5 (epsilon) (CCT5), mRNA
NM_004094.4	Homo sapiens eukaryotic translation initiation factor 2, subunit 1 alpha, 35 kDa (EIF2S1), mRNA
NM_014177.2	Homo sapiens chromosome 18 open reading frame 55 (C18orf55), nuclear gene encoding mitochondrial protein, mRNA
NM_006082.2	Homo sapiens tubulin, alpha 1b (TUBA1B), mRNA
NM_002568.3	Homo sapiens poly (A) binding protein, cytoplasmic 1 (PABPC1), mRNA
NM_004039.2	Homo sapiens annexin A2 (ANXA2), transcript variant 3, mRNA
NM_175066.3	Homo sapiens DEAD (Asp-Glu-Ala-Asp) box polypeptide 51 (DDX51), mRNA
NM_018492.2	Homo sapiens PDZ binding kinase (PBK), mRNA
NM_024636.3	Homo sapiens STEAP family member 4 (STEAP4), mRNA
NT_022517.18	Homo sapiens chromosome 3 genomic contig, GRCh37.p2 reference primary assembly
NM_006111.2	Homo sapiens acetyl-CoA acyltransferase 2 (ACAA2), nuclear gene encoding mitochondrial protein, mRNA
NM_001428.3	Homo sapiens enolase 1, (alpha) (ENO1), mRNA
NM_021130.3	Homo sapiens peptidylprolyl isomerase A (cyclophilin A) (PPIA), mRNA

### UBE2D3 Physically Interacts with hTERT

**Figure 2:**
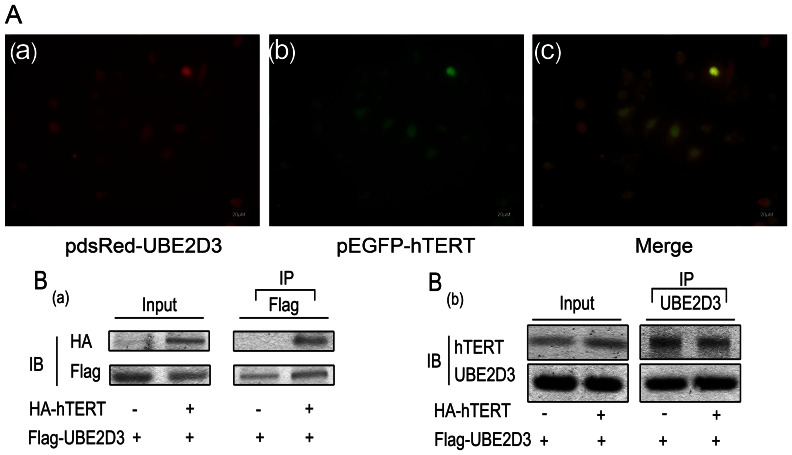
hTERT interacts with UBE2D3 proteins. (A)pEGFP-hTERT and pdsRed-UBE2D3 were co-transfected into MCF-7 cells. UBE2D3 with red fluorescent tag(A-a), hTERT with green fluorescent tag(A-b). The 3rd pic is the merge(A-c). From the picture above, we could indicate most of them express in the nucleus and the possibility of interaction between them in space. (B-a)HA-tagged hTERT and/or FLAG-tagged UBE2D3 plasmids were co-transfected into HEK293T cells as indicated. At 24 h after transfection, whole-cell lysates were isolated. Cell lysates were immunoprecipitated with anti-FLAG and the hTERT proteins in the complex identified with immunoblotting with anti-HA (top panel). UBE2D3 and hTERT protein expression was demonstrated with direct immunoblotting(IB) of cell lysates with HA antibody and FLAG antibody (left panel or input), respectively. (B-b)The above plasmids cotransfected into HEK293T cells as indicated. Cell lysates were immunoprecipitated with UBE2D3 and the hTERT proteins in the complex identified with immunoblotting with anti-hTERT (top panel). UBE2D3 and hTERT protein expression was demonstrated with direct immunoblotting of cell lysates with UBE2D3 and hTERT antibody (left panel or input), respectively. All experiments were repeated 3 times with similar results.

We next used a biochemical approach to validate the interaction between hTERT and UBE2D3. Plasmids pGBKT-hTERT and pGADT-UBE2D3 were transfected into Gold and Y187 yeast competent cells, respectively. Blue colonies were observed on QDO/X/A plate, demonstrating interaction between hTERT and UBE2D3, whereas no colony was observed on the negative control plate (data not shown). Next, plasmids pEGFP-hTERT and pdsRed-UBE2D3 were co-transfected into MCF-7 cells. Fluorescent co-localization of both proteins was detected primarily in the nucleus (Figure 2A). Given the results above, we conclude that hTERT might interact with UBE2D3. To validate this possibility, HEK293T cells were transfected with Flag-UBE2D3 and HA-hTERT, and then subjected to co-immunoprecipitation (Co-IP). HEK293T cells were immunoprecipitated with UBE2D3 or Flag antibody, and anti-hTERT or anti-HA antibodies were used in western blotting to determine the presence of hTERT in the UBE2D3 immunoprecipitates. As shown in Figure 2B, we found that UBE2D3 did indeed bind to hTERT.

### Knockdown of UBE2D3 Increases hTERT and Cyclin D1 Protein Expression

**Figure 3:**
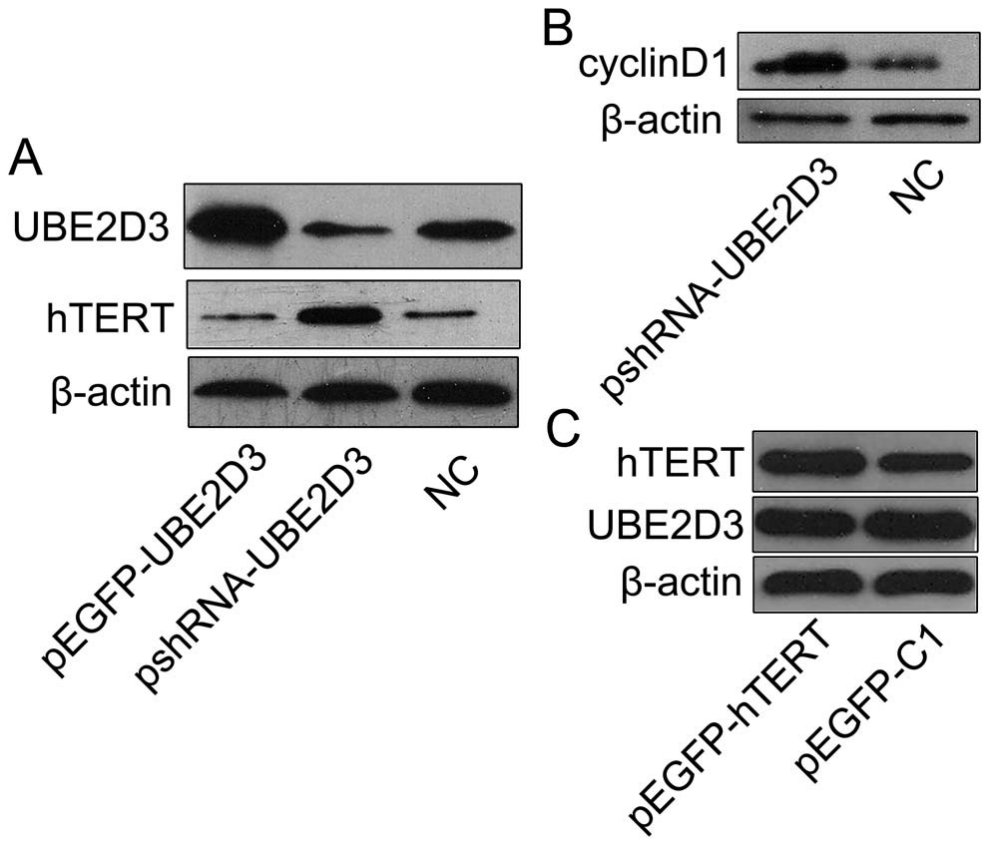
The detection of protein(UBE2D3, hTERT, cyclin D1, β-actin) expressions were illustrated. (**A**) Western blotting analysis showing the effect of overexpression and knockdown of UBE2D3 on UBE2D3 and hTERT levels in MCF-7 cells. Control cells were transfected with negative control shRNA. (**B**) Western blotting analysis showing the effect of knockdown of UBE2D3 on cyclin D1 levels in MCF-7 cells. (**C**) Western blotting analysis showing the effect of overexpression of hTERT on UBE2D3 and hTERT levels in MCF-7 cells. Experiments were repeated 3 times with similar results.

To evaluate whether down-regulation of UBE2D3 correlates with hTERT and cyclin D1 protein expression levels in MCF-7 cells, we alternately overexpressed and underexpressed UBE2D3 by transfecting pEGFP-UBE2D3 and pshRNA-UBE2D3, respectively, into MCF-7 cells. Figure 3A and 3B indicates that expression of hTERT and cyclin D1 in MCF-7 cells increased when UBE2D3 was down-regulated, leading us to speculate that the increase of hTERT resulted from the repression of UBE2D3-mediated ubiquitination. No changes in hTERT and cyclin D1 levels were observed when UBE2D3 was overexpressed (Figure 3A). Moreover, expression levels of UBE2D3 were unchanged when hTERT was overexpressed (Figure 3C).

### UBE2D3 was Involved in the MCF-7 Cell Cycle and Proliferation

**Figure 4:**
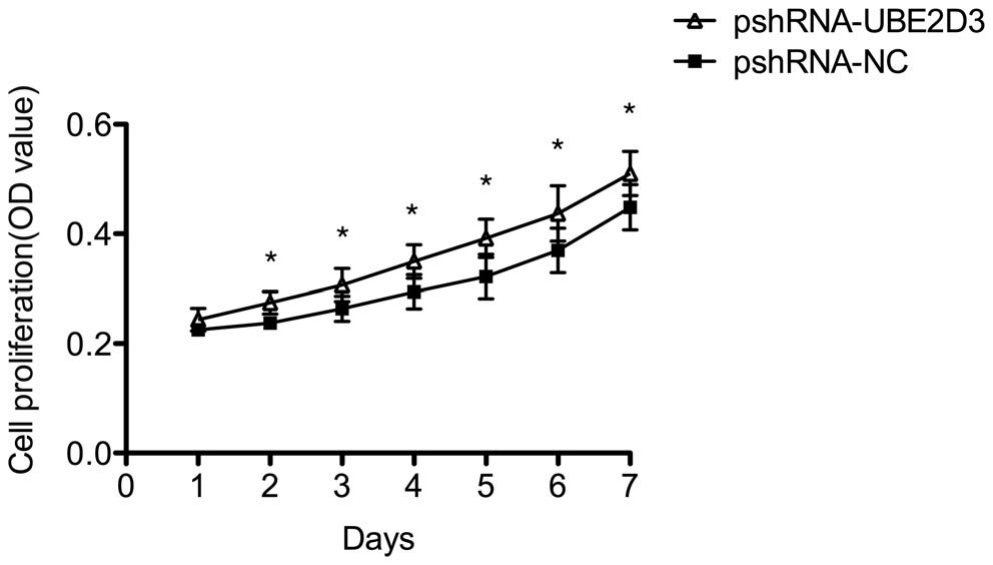
The MCF-7 cells proliferation were illustrated. After MCF-7 cells were transfected with pshRNA-UBE2D3, cell proliferation was examined by CCK-8 assay. The results were presented as the Means±SD of three independent experiments. *p<0.05.

Downregulation of UBE2D3 by transfection with pshRNA-UBE2D3 increased the proportion of MCF-7 cells in the S phase, representing an acceleration in the G1/S phase transition. Transfection with pshRNA-UBE2D3 resulted in a 54% decrease in the number of MCF-7 cells in the G1 phase and 1.28-fold increase in the number of MCF-7 cells in the S phase (data not shown). This phenomenon may be due to increased hTERT and cyclin D1 expression after down-regulation of UBE2D3. Next, the effect of UBE2D3 on the viability of MCF-7 cells was determined using a CCK-8 assay. MCF-7 cells were transfected with pshRNA-UBE2D3 for different time periods (1, 2, 3, 4, 5, 6 and 7 days). A time-dependent increase in cell viability was observed after repression of UBE2D3. The CCK-8 assay showed that after silencing of UBE2D3, there was a significant increase (*P<0.05*) in cell proliferation compared with the negative control (Figure 4).

### Down-regulation of UBE2D3 Enhanced Telomerase Activity

**Figure 5:**
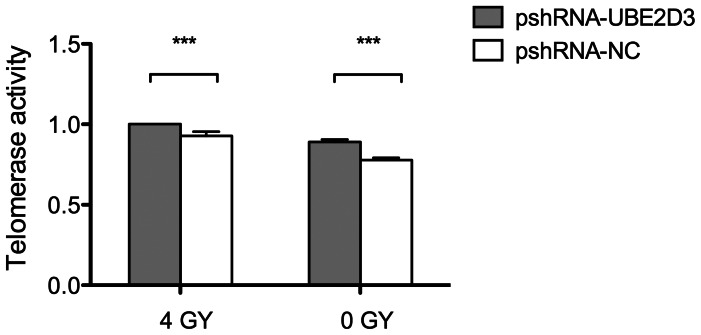
The MCF-7 cells telomerase activity was illustrated. MCF-7 cells were transfected with pshRNA-UBE2D3. After 48 hr, PCR-Elisa assay was used to detect telomerase activity. Furthermore, telomerase activity was also measured by 4 GY X-rays after transfection with pshRNA-UBE2D3 and negative control, which showed that MCF-7 cells treated with X-rays after transfection with pshRNA-UBE2D3 showed higher telomerase activity compared with transfection with pshRNA-UBE2D3 alone. Data represented Mean±SD of three independent experiments performed in triplicate. Error bars represent standard deviations.

Telomerase activity is regarded as the primary determinant of tumor cell radiosensitivity. To examine the effect of UBE2D3 on telomerase activity, we treated MCF-7 cells with pshRNA-UBE2D3 and negative control for 24 hr. Cell lysates were titrated between 0.001 and 2 mg protein per assay using a telomerase PCR-ELISA technique. MCF-7 cells transfected with pshRNA-UBE2D3 showed higher telomerase activity compared to negative control (*P*<0.05) (Figure 5). On the basis of these preliminary results, we treated MCF-7 cells with 4 GY X-ray after transfection with the above plasmids. MCF-7 cells treated with X-rays after transfection with pshRNA-UBE2D3 showed higher telomerase activity compared with transfection with pshRNA-UBE2D3 alone, suggesting that UBE2D3-induced elevation of hTERT activity could be enhanced by radiation treatment.

### Down-regulation of UBE2D3 Weakened MCF-7 Cells Radiosensitivity

**Figure 6:**
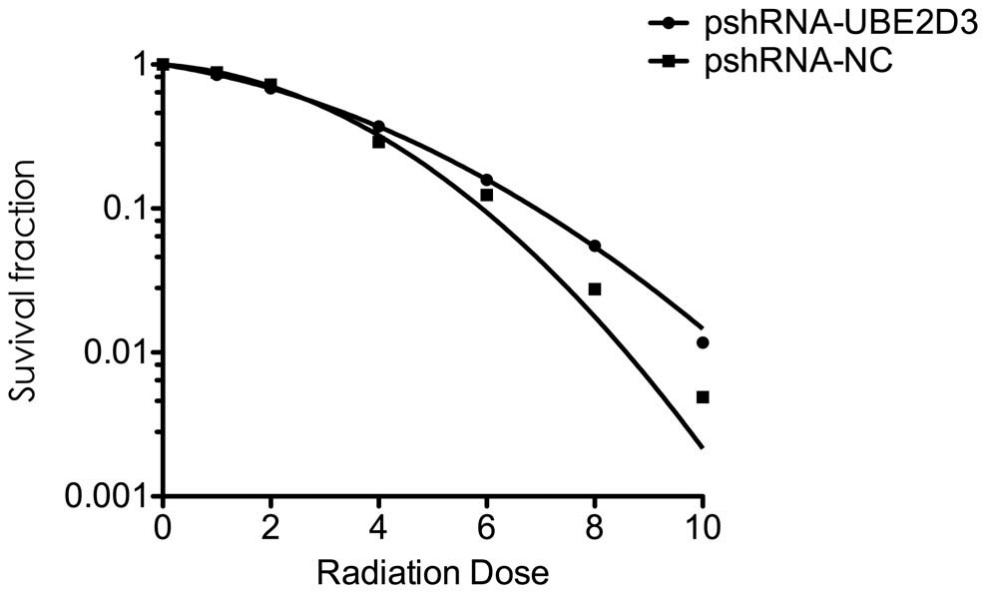
The MCF-7 cells radiosensitivity detection were illustrated when exposed to irradiation, depending on doses in GY, MCF-7 cells transfected with pshRNA-UBE2D3 showed reductions of clonogenic survival compared to negative control. Each group of cells were irradiated at the dose point of 0, 1, 2, 4, 6, 8, 10 GY respectively. After 14 days of incubation, the colonies were fixed and stained. Those colonies containing >50 cells were scored as viable colonies. The data were fit into the linear-quadratic model, and survival curve of each group were demonstrated by Graphpad prism 5.0 software. Each experiment was done at least three times in triplicate wells.

After counting clones, the survival curves were plotted to evaluate the radiobiological parameters of each group. Compared to the negative control, the survival fractions of the pshRNA-UBE2D3 group were much higher at each point in MCF-7 cells. Figure 6 shows that down-regulation of UBE2D3 reduced the radiosensitivity of MCF-7 cells. Similar results were observed in lung adenocarcinoma A549 cells (data not shown). Plating efficiency (PE) and survival fraction (SF) were calculated.

## Discussion

Here, we first performed Y2H to screen for hTERT-interacting proteins. We found evidence implicating UBE2D3 as a modulator of MCF-7 cell radiosensitivity by regulating hTERT and cyclin D1 protein expression. It is well established that telomerase activity requires the presence of the hTR and hTERT subunits. The present study of the relationship between hTERT and radiosensitivity indicates that in tumor cells, higher telomerase activity correlates with poorer radiosensitivity. Moreover, telomere length-independent mechanisms of telomerase activation may also be important [22]–[25]. In the light of our previous research, hTERT activity can be significantly repressed using Reverse Transcriptase Inhibitors (ATZ) or by transfection with pshRNA-hTERT, which increases cell radiosensitivity [11]. Other studies have shown that inhibition of the telomerase subunit hTERT increases radiosensitivity of tumor cells, causing cell death through inhibition of DNA double-strand damage repair mechanisms. Collectively these studies indicate that telomerase might be involved in DNA repair and chromosome healing [26].

As a broad spectrum of tumor molecular biomarkers, several transcription factors, including some oncogene and tumor suppressor gene products, are able to affect the hTERT transcription. hTERT promoter transcriptional activity is significantly associated with hTERT mRNA expression. For example, studies have shown that c-Myc and Sp1 can bind to the core hTERT promoter and increase hTERT mRNA levels [27]. Moreover, our previous study showed that a chimeric hTERT promoter containing 6 repeat CArG elements had an optimal radiation response compared with other chimeric promoters containing different numbers of CArG elements [28].

While most studies have focused on regulation of hTERT at the transcriptional level, our study has identified post-translational regulation of hTERT, through the interaction of the UBE2D3 with hTERT. Ubiquitination and degradation of proteins is one of the important intracellular post-translational modifications. Recently, the E2 enzymes are drawing the attention of researchers due to their perceived roles in the degradation of crucial regulatory molecules like IkB, TP53, and MDM2 [29], [30].

However, the functional role of E2 family members, including UBE2D3, in different kinds of tumors remains controversial. For example, Okamoto *et al.* showed that E2 enzyme gene UbcH10, is highly expressed in various human primary tumors compared with their corresponding normal tissues and that UbcH10 has an ability to promote cell growth and transformation. In contrast, UBE2D3 expression is lower in tumors than their corresponding normal tissues [31].In a previous study, we determined UBE2D3 expression of 30 cases of breast cancer patients and 20 cases of normal tissue through immunohistochemical methods, the results of which confirmed the trend observed on the genetic level.

Our current study has shown that down-regulation of UBE2D3 results in the accumulation of hTERT, while transfection with pshRNA-UBE2D3 combined with radiation treatment results in higher hTERT activity than radiation treatment alone. UBE2D3 was shown to regulate MCF-7 cells radiosensitivity through modulation of hTERT expression and activity, the mechanism of which may be involve hTERT ubiquitination.

Inhibition of MCF-7 UBE2D3 expression by shRNA in MCF-7 cells resulted in increased G1/S phase transition and accelerated MCF-7 cell proliferation. Hattori *et al*. overexpressed cyclin D1 to mimic the effect of transfection with pshRNA-UBE2D3, and confirmed that cyclinD1 was an essential downstream target of UBE2D3 [32]. Cyclin D1 is a key protein in regulation of the G1 phase, and its overexpression results in G1/S checkpoint disorders. Knockdown of UBE2D3 in SLUG-deficient human breast cells increased cyclin D1 levels, and stimulated proliferation and invasiveness of these cells [33]. It has also been reported that telomerase might promote cell proliferation by modulating expression of growth-controlling genes, such as EGFR, FGF and IL-1Ra [34]. Moreover, cyclin D1 overexpression results in inhibition of its ubiquitination, and it is degraded mainly via the 26S proteasome in a ubiquitin-dependent manner [35]. In addition, overexpression of cyclin D1 is associated with radioresistance via a mechanism involving the AKT/GSK3β/cyclin D1/Cdk4 pathway [36].

Our confirmation that hTERT and cyclin D1 mediated radiosensitization in a UBE2D3-dependent manner sheds light on the relationship between telomerase regulation and radiosensitivity. Our data imply that the abundance of cyclinD1 could accelerate G1 to S phase transition, reducing the number of cells surviving after injury, and leading to activation of DNA damage detection, increased DNA repair and radioresistance. The observed radioresistance after elevation of hTERT expression levels and activity were similar to the data obtained after cyclin D1 overexpression. The up-regulation of telomerase expression level and its activity may be a reaction to DNA damage induced by irradiation, and may be one of the mechanisms involved in radiation resistance in tumor cell lines.

Although the Y2H assay is a well-established method for finding novel protein-protein interactions, it is not very specific and routinely produces false positives. We chose UBE2D3 as the aim of our study, based on its interaction with hTERT. Although UBE2N and UBE2D3 are members of the E2 family, their E2 specificity will be investigated in detail in our research. In future studies, we will clarify their role in ubiquitination in more detail.

Taken together, we have shown that MCF-7 cells transfected with pshRNA-UBE2D3 have increased radioresistance. We have confirmed that repression of UBE2D3 increases both telomerase expression levels and activity. Our data suggest that depletion of UBE2D3 mediates cell proliferation by up-regulation of cyclinD1 expression and hTERT activity. Although our research group observes the similar results on radiosensitivity in A549 cells, the mechanisms need to be deeply investigated. To the best of our knowledge, this is the first report to demonstrate that inhibition of UBE2D3 decreases MCF-7 cell radiosensitivity, and these results provide new insights into the UBE2D3-hTERT pathway. Our data may also represent a starting point for new therapeutic strategies based on the assessment of UBE2D3, hTERT and cyclinD1 expression levels as predictors of radiotherapy outcome.
